# Emotional and Cognitive “Route” in Decision-Making Process: The Relationship between Executive Functions, Psychophysiological Correlates, Decisional Styles, and Personality

**DOI:** 10.3390/brainsci14070734

**Published:** 2024-07-22

**Authors:** Davide Crivelli, Carlotta Acconito, Michela Balconi

**Affiliations:** 1 International Research Center for Cognitive Applied Neuroscience (IrcCAN), Faculty of Psychology, Università Cattolica del Sacro Cuore, 20123 Milan, Italy; carlotta.acconito1@unicatt.it (C.A.); michela.balconi@unicatt.it (M.B.); 2 Research Unit in Affective and Social Neuroscience, Department of Psychology, Università Cattolica del Sacro Cuore, 20123 Milan, Italy

**Keywords:** decision-making, personality, executive functions, EEG, emotions, decisional style, heart rate

## Abstract

Studies on decision-making have classically focused exclusively on its cognitive component. Recent research has shown that a further essential component of decisional processes is the emotional one. Indeed, the emotional route in decision-making plays a crucial role, especially in situations characterized by ambiguity, uncertainty, and risk. Despite that, individual differences concerning such components and their associations with individual traits, decisional styles, and psychophysiological profiles are still understudied. This pilot study aimed at investigating the relationship between individual propensity toward using an emotional or cognitive information-processing route in decision-making, EEG and autonomic correlates of the decisional performance as collected via wearable non-invasive devices, and individual personality and decisional traits. Participants completed a novel task based on realistic decisional scenarios while their physiological activity (EEG and autonomic indices) was monitored. Self-report questionnaires were used to collect data on personality traits, individual differences, and decisional styles. Data analyses highlighted two main findings. Firstly, different personality traits and decisional styles showed significant and specific correlations, with an individual propensity toward either emotional or cognitive information processing for decision-making. Secondly, task-related EEG and autonomic measures presented a specific and distinct correlation pattern with different decisional styles, maximization traits, and personality traits, suggesting different latent profiles.

## 1. Introduction

The term decision-making could be defined as a pivotal skill or instrumental ability that allows individuals to discern a suitable course of action from a wide range of options [1,2,3]. Indeed, it refers to a goal-oriented process linked to executive functions (EFs) [1,4]. Encompassing high-level and goal-directed cognitive skills controlling emotion, thinking, and action, EFs are categorized as cold or hot based on emotional involvement and the affective/motivational connotation of the decisional situation [5]. Specifically, hot EFs involve affective aspects of EFs, such as information processing related to reward, emotion, and motivation, while cold EFs are linked to cognitive information-processing, involving flexibility, inhibition, working memory, planning, and organizing. Starting from these premises, cold EFs require more conscious control, mental effort and critical analysis compared with hot EFs [6,7]. Both EF types rely on partly overlapping brain regions, representing higher-level self-regulatory processing, often collaborating in real-world problem-solving [5].

Traditionally, decision-making studies have focused solely on the cognitive aspects (cold EFs), considering logical inference and cos–benefit calculation, but recent research has emphasized the emotional component in decision-making (hot EFs), crucial in ambiguous, uncertain, and risky situations [8,9,10,11,12]. Also, bidirectional influences between cognitive and emotional evaluations have been discussed [13,14,15]. The presence of a cognitive and emotional component in decision-making processes has led to the development of the so-called “dual decision-making model” [16], with two distinct systems that alternately compete and cooperate to arrive at a decision. Specifically, the dual decision-making model posits two interacting, though competing, systems [14,17,18]: System 1 and System 2. System 1 is the intuitive, automatic, unconscious and effortless system, referring to the emotional component of information processing and fast processes supporting intuitive decisional strategies. On the other hand, System 2 is a controlled, deliberate, conscious, and effortful system linked to the cognitive components and to deliberative and controlled decision-making strategies.

Despite recognizing the interplay between cognitive and emotional processes, there’s limited research on individual differences related to information processing for decisions, decisional styles, and psychophysiological profiles.

A neuroscientific multi-methodological approach, aiming to explore both explicit and implicit aspects of decision-making [4,19], can enhance understanding of subjective determinants, neurofunctional correlates, and core constituents of decisional processes. This involves investigating explicit components through ecological tasks and complementing them with physiological markers like electroencephalography (EEG) and autonomic activity [20,21]. Indeed, EEG monitors brain electrical activity, interpreting changes in frequency bands as indicators of cognitive load and mental effort [19,22,23], cognitive control and monitoring [24,25], and affective regulation [24,26]. Conversely, changes in physiological arousal and parasympathetic activity as measures via electrodermal measures (Skin Conductance Level and Response—SCL and SCR) and cardiovascular measures (Heart Rate and Heart Rate Variability—HR and HRV) mirror stress levels and emotional commitment during decision-making tasks [21,27].

Finally, a comprehensive multi-method approach for understanding subjective differences in information processing for decision-making should include the exploration of personality traits and styles. Numerous studies have established a connection between personality and higher cognitive functions, including decision-making [3,28]. For example, Emotional Stability and Conscientiousness in the Big Five model are positively linked to higher cognitive functions [29]. Also, optimal EF correlates positively with Rational and Intuitive decision-making styles, as measured by the General Decision-Making Style (GDMS) inventory, and negatively with Dependent and Avoidant styles [30,31]. Personality traits are also associated with individual tendencies in emotional or cognitive information processing. In the Big Five model, Openness and Conscientiousness are linked to a preference for cognitive processing, while Emotional Stability and Extraversion are correlated with emotional information processing [32,33,34]. By examining GDMS factors, instead, Rational and Dependent decision-making styles align with cognitive information processing, whereas the Spontaneous style aligns with emotional information processing [30]. Again, drawing parallels between the Big Five traits and GDMS styles reveals correlations [35,36], such as Rational and Intuitive styles aligning with Openness and Conscientiousness. The Intuitive style has also been correlated with high Agreeableness and Extroversion, suggesting a connection between Relational and Intuitive styles and cognitive information processing.

Furthermore, various personality traits and decision-making styles may exhibit associations with specific EEG frequency bands and autonomic patterns. In terms of autonomic activity, research has shown a positive correlation between HRV and personality traits linked to flexible decision-making, such as Conscientiousness and Emotional Stability [37]. Regarding EEG, while no previous studies—to the best of our knowledge—have directly linked EEG profiles to decision-making styles, it is plausible to hypothesize associations based on the functional significance of electrophysiological oscillations. For instance, considering that theta band activity indicates cognitive control, working memory, and emotion regulation [24,25,26,38,39], this EEG pattern might be associated with the Rational decision-making style. This style characterizes individuals who strive to select the best possible choice by analyzing multiple alternatives. Similarly, the beta band, reflecting workload management and cognitive resource regulation [22,23,40,41], could connote the decision-making process of individuals actively searching for, evaluating, and choosing between alternatives.

Within this methodological framework, our pilot explorative study employed a multi-methodological approach, collecting behavioral, EEG, autonomic, and self-report data related to decision-making using non-invasive wearable devices. The goal was to explore the link between individual propensity toward emotional or cognitive information processing in decision-making, individual personality and decisional traits, and EEG and autonomic correlates during a realistic decision task.

Concerning the relationship between individual propensity toward emotional or cognitive information processing and individual differences, we anticipated that a preference for emotional information processing would correlate with greater Emotional Stability, while a preference for cognitive information processing would show associations with Openness and Conscientiousness traits. Furthermore, we expected correlations between Rational and Dependent GDMS styles and cognitive information processing, as well as Spontaneous style, with a preference for emotional information processing. Secondly, regarding individual profiles and EEG correlates, we hypothesized that synchronization of the theta band, reflecting information integration and enhanced cognitive control, might be associated with a comprehensive search for information mirrored by greater Rational GDMS scores. Finally, considering personality profiles and autonomic responses, we hypothesized that Conscientiousness and Emotional Stability traits would correlate with higher HRV, which is indicative of self-regulation and inhibitory control. Indeed, these traits align with the ability to resist impulses, pursue goal-directed behaviors, and adapt to change, striving for the highest standards.

## 2. Materials and Methods

### 2.1. Sample

Twenty-four adult individuals (13/11 M/F; M_age_ = 35.3, SD_age_ = 11.7) without any specific area of professional specialization gave their written informed consent to participate in the pilot explorative study. Recruitment was managed via consecutive sampling. No compensation was provided for study participation. The exclusion criteria were a history of psychiatric or neurological disease, ongoing therapy with psychoactive drugs, clinically relevant distress level, altered global cognitive functioning, and occurrence of major stressful life events in the past six months. All participants had normal or corrected-to-normal vision and a minimum schooling level of eighteen years, as all participants had master’s degrees. Some of the professionals also had higher qualifications, such as a doctoral title. Post hoc power analysis was run to compute the achieved power and further test the robustness of the reported findings. The analysis—based on an average obtained effect size of 0.56, α = 0.05, and N = 24—estimated an average achieved power equal to 0.84 (GPower version 3.1.9.7; [42]).

The experimental study was conducted in compliance with the Helsinki Declaration (2013), and the entire protocol was approved by the Ethics Committee of the Department of Psychology at the Università Cattolica del Sacro Cuore.

### 2.2. Procedure

Participants were introduced to the study procedures and task, presented with informed consent, and asked to fill a survey including self-report measures and questionnaires (duration ≈ 20 min). Then, participants were asked to wear non-invasive devices for EEG and autonomic measures to collect physiological data at rest and during the experimental task.

#### 2.2.1. Behavioural Data Acquisition

The experimental task, devised to investigate individual propensity toward emotional and cognitive information processing in real-life decision-making situations, was administered via an experiment-management platform (PsyToolkit, version 3.4.4; [43]). PsyToolikt is a free web-based resource specifically designed for running online questionnaires and cognitive psychological experiments, collecting behavioral and response time (RT) data in millisecond timing precision.

Specifically, they were asked to immerse themselves in four scenarios presenting realistic situations in which they had to make a difficult and affectively engaging decision. Each decisional situation was then followed by four statements to which participants had to provide a response on a five-point Likert scale (1 = “totally disagree”, 5 = “totally agree”). Two of the statements were designed to implicitly stress affective connotations of the decisional process and prompt an emotional information-processing route to decisional outcomes, while the other two implicitly stressed rational connotations of the decisional process and prompted a cognitive information-processing route to decisional outcomes. Please see Supplementary Material S1 for additional methodological notes concerning the task and its validation and for an exemplifying scenario with related statements.

For each scenario, response scores and RTs were collected and used to compute two indices: the Emotional Component Propensity index (ECP_i_) and the Cognitive Component Propensity index (CCP_i_). We opted to incorporate RTs into the calculation of ECP_i_ and CCP_i_ to obtain finer-grained assessment metrics that could mirror not only the outcome of a decisional process but also the mental load imposed by such a process. Indeed, by weighing participants’ responses on their RTs, it is possible to account for the level of cognitive effort that connotes the appraisal and decision-making processes of each individual in each different situation. This approach assumes that shorter RTs indicate lower task-related effort and greater information-processing efficiency, which may be indicative of the preferred information-processing route in decision-making.

The ECP_i_ and CCP_i_ were calculated considering the response scores and RTs given to statements with, respectively, emotional vs. cognitive connotation across the four scenarios converted in a common decile scale as follows:

ECP_i_ = (Resp_Emo-Org_ − Resp_Emo-Med_)/((RT_Emo-Org_ + RT_Emo-Med_)/2),

CCP_i_ = (Resp_Cog-Org_ − Resp_Cog-Med_)/((RT_Cog-Org_ + RT_Cog-Med_)/2),

where, in the ECP_i_ index, Resp_Emo-Org_ and Resp_Emo-Med_ stand respectively for decile response scores at emotion-connoted statements for organizational scenarios and medical scenarios, while RT_Emo-Org_ and RT_Emo-Med_ refer to mean RTs converted in a decile scale for emotion-connoted statements in organizational and medical scenarios. Similarly, the terms in the CCP_i_ equation refer to the same elements previously explained but are related to cognition-connoted statements.

Please see Supplementary Material S2 for the specifics on ECP_i_ and CCP_i_ calculations.

#### 2.2.2. Electrophysiological Data Acquisition

To collect electrophysiological data in a resting state and highlight task-related variations in EEG power, we used the Muse^TM^ headband (InteraXon Inc., Toronto, ON, Canada). This wearable EEG allows for the collection of EEG data from four electrodes positioned in the frontal (AF7 and AF8) and temporoparietal (TP9 and TP10) areas. While we acknowledge that EEG recording, as performed here, presents limitations in terms of spatial resolution and topographic mapping, our choice to use a wearable device was guided by the predominant intent to preserve the ecological validity of the setting and the low invasivity of physiological recording. We considered those latter aspects as primary with respect to the former (i.e., the density of the recording montage) since we did not aim at discussing anatomical-functional correlates of observed EEG responses. We do, instead, considered it crucial to maintain recording and task performance as naturally as possible.

The EEG data were gathered using the mobile app Mind Monitor and transferred via Bluetooth to an associated smartphone. Data were sampled at 256 Hz with a 50 Hz notch filter. The raw data, thanks to the Mind Monitor app, were also converted in real time in Power Spectral Density (PSD) via Fast Fourier Transformation, and PSD values were computed for each frequency band using 2 s epochs: delta (1–4 Hz), theta (4–8 Hz), alpha (8–13 Hz), beta (13–30 Hz), and gamma (30–44 Hz). Then, average PSD values were extracted for both baseline (eye-open and eyes-closed resting, duration: 120 s) and task. Finally, task-related variations in EEG power values were computed with Excel as follows:

TR_PSD_ = ((PSD_task_ − PSD_open-bl_)/PSD_open-bl_) × 100

Please see Supplementary Material S2 for specifics on the TR_PSD_ calculation.

#### 2.2.3. Autonomic Data Acquisition

A wearable device was also used to collect autonomic parameters at rest and during the task (X-pert^2000^; Schuhfried GmbH, Modling, Austria). Specifically, electrodermal (SCL, SCR) and cardiovascular (HR, HRV) measures were collected via a multipurpose sensor placed on the distal phalanx of the second finger of the non-dominant hand. Specifically, electrodermal activity was sampled at 40 Hz, and SCR was derived from SCL via an automated moving average approach. On the other hand, cardiovascular measures were sampled at 100 Hz, and HR was derived by fluctuations of blood volume per pulse data via an automated algorithm, while HRV was calculated as the standard deviation of inter-beat intervals. The inbuilt accelerometer was used to monitor hand movements to remove potential interference from the recording during analysis. Task-related variations in autonomic indices were computed as follows:

TR_AI_ = ((AI_task_ − AI_open-bl_)/AI_open-bl_) × 100

Please see Supplementary Material S2 for specifics on the TR_AI_ calculation.

#### 2.2.4. Self-Report Data Acquisition

Self-report data were collected using the General Decision-Making Style—GDMS [30,44], the Maximization Scale—MS [45], the 10-item Big Five Inventory—BFI [46], and the Five Facet Mindfulness Questionnaire—FFMQ [47].

The GDMS permits the investigation of the individual’s decision-making style, which can be defined as Rational, Intuitive, Dependent, Avoidant, and Spontaneous, depending on the score given along a five-point Likert scale (1 = strongly disagree, 5 = strongly agree), each subject to 25 items. The difference between these five styles is in the way decisions are made: in the Rational style, all alternatives and their consequences are considered by researching as much information as possible; in the Intuitive style, global aspects are analyzed, and decisions are made based on intuitions; in the Dependent style, suggestions and advice are preferred; in the Avoidant style, decisions tend not to be made; and in the Spontaneous style, decisions are made as quickly as possible.

The MS is a 13-item questionnaire which permits to identify individual differences related to the tendency to maximize one’s choice or to be satisfied with a decision considered good enough. The questionnaire allows to compute three subscale scores: Alternative Search, mirroring the tendency to look for alternative options or solutions when involved in decisions; Decision Difficulty, mirroring subjective effort and frustration when involved in decision-making; and High Standards, mirroring the strive, desire, and active search for the best option when involved in decision-making.

The BFI, on the other hand, allows for an individual’s personality to be assessed along several five subscales (Extroversion, Agreeableness, Conscientiousness, Emotional Stability, and Openness), depending on the score given to the 10 items assessed with a five-point Likert scale (1 = strongly disagree, 5 = strongly agree). Specifically, Extroversion relates to being active and enthusiastic, Agreeableness to being pleasant and warm, Conscientiousness to being organized and self-disciplined, Emotional Stability to being calm, stable, and equilibrated, and Openness to being creative and open to experiencing novel things.

Finally, the FFMQ is an instrument devised to measure five different aspects of mindfulness by rating 39 items on a five-point Likert scale (1 = never true, 5 = always true). The five different aspects are Observing (the ability to note sensations, feelings, thoughts, etc.), Describing (the ability to label and represent verbally feelings, emotions, and thoughts), Acting with Awareness (individual awareness of own actions and behavior), Not judging the inner experience (the propensity to accept sensation, feelings, emotions and thoughts as they are), and Not reacting to the inner experience (the ability to enact actual responses to internal and external stimuli instead of automatic reactions).

### 2.3. Data Analyses

Data analyses in this study were planned according to two sequential steps and performed using Jamovi software (v. 2.3.28). The first one explored the pattern of correlations between the propensity toward emotional and cognitive information-processing routes in decision-making and individual traits via Pearson correlation coefficients. In the second one, we explored their correlations with physiological activity associated with decision-making performance. Specifically, in the first step, ECP_i_ and CCP_i_ were correlated with scale scores of the GDMS, MS, BFI, and FFMQ. In the second step, Pearson correlation coefficients were computed between ECP_i_, CCP_i_, GDMS scores, MS scores, BFI scores and FFMQ scores, and TR_PSD_ for EEG bands (electrodes: AF7, AF8, TP9, TP10; EEG bands: delta, theta, alpha, beta, and gamma) or TR_AI_ for autonomic indices (SCL, SCR; HR, HRV).

Assumptions of normality for distributions of data entering correlation analyses were checked by computing asymmetry and kurtosis values and comparing them against the reference cut-off, defined as ±1.5 for asymmetry and ±3 for kurtosis. While the reference literature in psychometrics suggests considering a data distribution to be normal if asymmetry is between ±2 and kurtosis is between ±7 (see, as examples, [48,49]), in defining our cut-offs, we opted for stricter and more conservative rules. Also, the outcomes of correlation analyses were checked against the false discovery rate by applying the Benjamini–Hochberg procedure [50] to minimize the risk of reporting and interpreting results biased by potential multiple testing errors. Full correlation matrices are included in Supplementary Material S3.

## 3. Results

### 3.1. Step One

**Figure 1:**
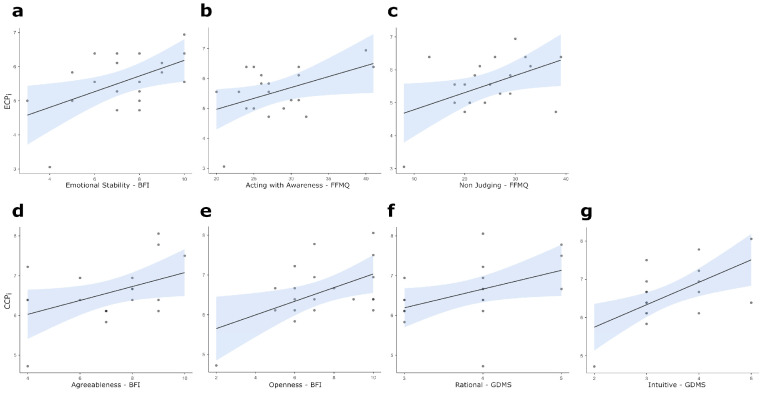
Scatterplots for statistically significant correlations between the following: (**a**) ECP_i_ and Emotional Stability; (**b**) ECP_i_ and Act with Awareness; (**c**) ECP_i_ and Non-Judging; (**d**) CCP_i_ and Agreeableness; (**e**) CCP_i_ and Openness; (**f**) CCP_i_ and Rationale score; (**g**) CCP_i_ and Intuitive score. The straight lines represent the global linear trends, while the shades represent their 95% confidence intervals. Light blue profiles on top and at the right of each scatterplot represent the density plots for correlated variables. Notes: ECP_i_ = Emotional Component Propensity index; CCP_i_ = Cognitive Component Propensity index; BFI = Big Five Inventory; FFMQ = Five Facet Mindfulness Questionnaire; GDMS = General Decision-Making Style.

Correlation analyses computed between ECP_i_, CCP_i_ and scale scores from GDMS, MS, BFI, and FFMQ highlighted significant positive correlations between the ECP_i_ and the BFI EmotionalStability score (*r* = 0.524, *p* = 0.018, Figure 1a), and the FFMQ ActWithAwareness (*r* = 0.458, *p* = 0.042, Figure 1b) and NonJudging (*r* = 0.467, *p* = 0.038, Figure 1c) scores. Again, analyses highlighted significant positive correlations between the CCP_i_ and the BFI Agreeableness (*r* = 0.450, *p* = 0.046, Figure 1d) and Openness (*r* = 0.522, *p* = 0.018, Figure 1e) scores, and the GDMS Rationale (*r* = 0.447, *p* = 0.048, Figure 1f) and Intuitive (*r* = 0.603, *p* = 0.005, Figure 1g) scores. No other correlation coefficient was found to reach the statistical threshold.

### 3.2. Step Two

**Figure 2:**
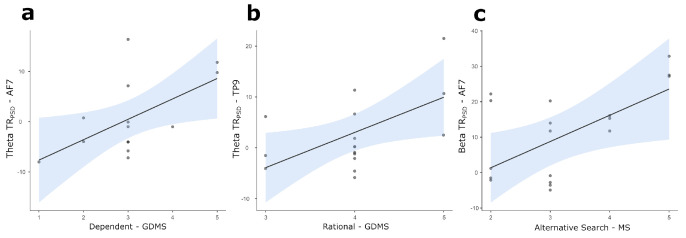
Scatterplots for statistically significant correlations between: (**a**) Dependent score and theta TR_PSD_ in AF7; (**b**) Rational score and theta TR_PSD_ in TP9; (**c**) Alternative Search and beta TR_PSD_ in AF7. The straight lines represent the global linear trends, while the shades represent their 95% confidence intervals. Light blue profiles on top and at the right of each scatterplot represent the density plots for correlated variables. Notes: TR_PSD_ = task-related changes in EEG power density; GDMS = General Decision-Making Style; MS = Maximization Scale.

Correlation analyses highlighted positive significant correlations between the GDMS Dependent score and theta TR_PSD_ in AF7 (*r =* 0.574, *p* = 0.032, Figure 2a) and the GDMS Rational score and theta TR_PSD_ in TP9 (*r =* 0.554, *p* = 0.032, Figure 2b). Again, analyses highlighted a positive significant correlation between the MS AlternativeSearch score and beta TR_PSD_ in AF7 (*r =* 0.623, *p* = 0.030, Figure 2c).

**Figure 3:**
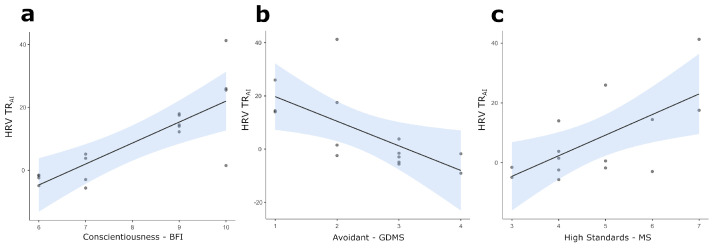
Scatterplots for statistically significant correlations between the following: (**a**) Conscientiousness and HRV TR_AI_; (**b**) Avoidant score and HRV TR_AI_; (**c**) High Standards and HRV TR_AI_. The straight lines represent the global linear trends, while the shades represent their 95% confidence intervals. Light blue profiles on top and at the right of each scatterplot represent the density plots for correlated variables. Notes: TR_AI_ = task-related changes of autonomic indices; HRV = Heart Rate Variability; HR = Heart Rate; BFI = Big Five Inventory; GDMS = General Decision-Making Style; MS = Maximization Scale.

Furthermore, analyses highlighted significant positive correlations between the BFI Conscientiousness score and HRV TR_AI_ (*r =* 0.787, *p* = 0.001, Figure 3a). Again, analyses highlighted a significant negative correlation between the GDMS Avoidant score and HRV TR_AI_ (*r =* −0.617, *p* = 0.025, Figure 3b) and a significant positive correlation between the MS HighStandards score and HRV TR_AI_ (*r =* 0.661, *p* = 0.014, Figure 3c).

No other correlation coefficient was found to reach the statistical threshold.

## 4. Discussion

This exploratory study yielded two main outcomes. Firstly, different personality traits and decisional styles showed significant and specific correlations with individual propensity toward either emotional or cognitive information processing for decision-making. Secondly, task-related EEG and autonomic measures showed distinct correlation patterns with different decisional styles, maximization traits, and personality traits.

As for the first main point, individuals with a greater propensity for emotional information processing in decision-making showed higher EmotionalStability traits, as well as greater ActingWithAwareness and NotJudging FFMQ scores. On the other hand, those inclined toward cognitive information processing presented higher Agreeableness and Openness traits. Additionally, they tended to prefer both a Rational and Intuitive decision-making style. These associations align with existing evidence based on the Big Five model, linking Openness and EmotionalStability traits to, respectively, cognitive and emotional information-processing routes [32,33,34]. Moreover, the Agreeableness trait correlates with effective self-regulation and reduced impulsivity [51], features that likely connote a rational and logical decision-making approach.

In addition, individuals inclined toward an emotional route in decision-making obtained higher ActingWithAwareness and NotJudging FFMQ scores. Although no prior research has explicitly discussed this association, we propose that it could reflect a common grounding of mindfulness traits and the tendency to approach decision-making emotionally on self-awareness/regulation skills. We, nonetheless, acknowledge that this potential interpretation is tentative and requires further and specific testing, making it a subject for future empirical investigations.

Again, the observed association between a propensity for cognitive information processing and a preference for a rational or intuitive decision-making style aligns with existing theorization on the meaning and correlates of GDMS factors [30]. The rational style involves searching for information and logically evaluating alternatives, while the intuitive style is characterized by attention to detail and reliance on intuitions. Despite the explicit vs. partly implicit use of information, both styles then ground on systematic collection of information for guiding decision processes, as well as on their integration and evaluation. This approach—involving methodical assessment of situations, opportunities, and constraints—resonates with the preference for a cognitive route in decision-making, characterized by a classically rational and logical way of appraising situations and making decisions.

Taken together, the above-discussed first set of evidence suggests that individuals who highly rely on an emotional route to decision-making are those connoted by greater affective balance and stress management skills, besides greater awareness and acceptance of their own behavior, thoughts, and affects. An optimal relationship with one’s own affective experiences might represent the key to justifying individual propensity toward emotional components even in decision-making.

As for the second main point, task-related theta indices over frontal sites correlated with greater propensity toward a Dependent decision-making style, while theta activity over posterior sites correlated with greater Rational GDMS scores. Greater beta TR_PSD_, instead, showed positive correlation with AlternativeSearch MS scores. Differently, greater HRV TR_AI_ correlated with greater Conscientiousness BFI scores, greater HighStandards MS scores, and lower Avoidant GDMS scores.

Notably, this second set of findings partially supports the related hypotheses. While task-related modulations of theta activity were indeed associated with the Rational GDMS style, they also correlated with the Dependent style. As noted in the opening sections, no previous studies have directly explored the link between EEG modulations and decision-making styles. Yet, the correlation between a rational decision-making approach, characterized by active information search and systematic appraisal of situations and consequences, and posterior task-related theta modulations aligns with evidence on the functional significance of theta oscillations over temporoparietal areas. Temporoparietal theta activity has, indeed, been linked to perspective-taking, prosocial choices, and social decision-making [52,53,54,55]. The complex social scenarios presented in the research likely required individuals to engage in perspective-taking and social reasoning, especially those who tend to search and appraise each available information and the consequence of their own choices.

Differently, the unexpected correlation between a Dependent decision-making style, characterized by actively seeking suggestions and advice from others, and slow EEG activity over frontal sites may be interpreted in the context of the relationship between this style and lower self-esteem, impaired self-regulation, and difficulties in carrying out autonomous reasoning processes [56]. The greater theta activity over frontal areas might mirror the heightened need for cognitive control and emotion regulation during the decision-making process in individuals scoring high on the dependent style.

The positive correlation of beta TR_PSD_ and MS AlternativeSearch scores, instead, might be interpreted based on the functional significance of beta activity over frontal areas. Beta oscillations are considered indicative of workload management and endogenous top-down regulation of attention and mental resources [23,40,41]. It is plausible that individuals who consistently and systematically seek and assess alternative options or solutions in decision-making scenarios may also dedicate more neural resources to a realistic decisional task.

Finally, our last hypothesis was partly confirmed by data in that both EmotionalStability and Conscientiousness showed significant correlations with autonomic indices but not as expected. Namely, only the Conscientiousness trait proved to be positively correlated with HRV values, consistent with the limited evidence base [37,57].

HRV indices also showed a positive correlation with MS HighStandards scores and a negative correlation with GDMS Avoidant scores. Provided that the avoidant decision-making style was associated with ineffective self-regulation and low self-esteem [44,56], the inverse correlation with HRV—a marker of vagal tone and self-regulation [21,58,59]—outlines a consistent profile of altered regulatory processes. Building on such interpretation, the positive correlation between HRV indices and HighStandards scores suggests preserved and efficient self-regulation skills in people who aim at maximizing the outcome of decisional processes, striving and actively searching for the best option available.

As a concluding point, we acknowledge that the present study, while discussing novel findings concerning the association between individual differences in terms of subjective propensity toward a cognitive vs. emotional route in decision-making, personality traits, decisional styles, and physiological correlates of the decisional performance, still represent a first pilot exploratory account of such relationships and present limitations. The actual sample size is limited, making this work a pilot study. Present correlation findings and related interpretations hint at interesting implications for the main research question that guided our work—i.e., whether subjective propensity toward using an emotional or cognitive information-processing route in decision-making, individual traits, and psychophysiological correlates of realistic decisional performance are associated, and which association pattern connote such relationships. Yet, they would benefit from replication and further testing on parallel or larger samples, especially the ones concerning the correlations with neurofunctional and autonomic markers of decisional performance, to check for their robustness and generalizability. Also, in the decisional task, only two settings (organizational/professional and healthcare/medical ones) were used as a frame for challenging and affectively engaging decisional scenarios. Future studies could try to collect data using different settings (e.g., emergency management, family/caregiving) to complement current observations and further test-related conclusions. In addition, data interpretation and theoretical-practical remarks could be further enriched by follow-up analyses with larger samples focusing on describing and profiling individuals showing particularly high vs. low propensity toward using an emotional/cognitive information-processing route in decision-making via clusterization and between-subjects comparisons or network analyses. Such additional explorations would plausibly provide interesting results and further food for thought.

## Data Availability

The data presented in this study are available on request from the corresponding author due to ethical reasons for sensitive personal data protection (requests will be evaluated according to the GDPR—Reg. UE 2016/679 and its ethical guidelines).

## Supplementary Materials

The following supporting information can be downloaded at https://www.mdpi.com/article/10.3390/brainsci14070734/s1, Supplementary Material S1: Additional methodological notes on the novel experimental task; Supplementary Material S2: Additional methodological notes on score calculations; Supplementary Material S3: Correlation matrices: behavioral, self-report, and psychophysiological data.
