# Supplementary material for: Emotional and Cognitive “Route” in Decision-Making Process: The Relationship between Executive Functions, Psychophysiological Correlates, Decisional Styles, and Personality
Source: Brain Sci. 2024 Jul 22;14(7):734. doi: 10.3390/brainsci14070734 (PMC11274958; doi:10.3390/brainsci14070734)
Supplement: Supplementary file 1 [file brainsci-14-00734-s001.zip › SupplMatS1_BrainSciences.pdf]

**Supplementary Material S1**

***Additional methodological notes on the novel experimental task***

The task is constituted by four decisional scenarios in which participants were asked to immerse themselves and make behavioural decisions, followed by four statements to which they have to express their degree of agreement. The scenarios presented realistic situation in which a difficult and affectively engaging decision has to be made. In two of them the decision was framed in a work-related setting, while in the other two the concerned a health-related medical setting. Such settings were chosen for their salience, their impact on arousal, and their potential for affective engagement.

All scenarios were created ensuring the ecological validity of the setting and the relevance of the decision-making processes. To check for such features, the scenarios have undergone validation by independent judges, who rated their ecological and construct validity, and their ability to promote engagement, as well as their realism, intelligibility, and clarity.

As for the scenarios, the statements that followed them have undergone validation by independent judges, who rated their plausibility, intelligibility, clarity, and the degree to which they conveyed the intended emotional vs. cognitive connotation of decision-making.

**Table S1.** Example of a realistic decisional scenarios with related statements and an indication of their primarily cognitive or emotional connotation

| Scenario | Questions |
|---|---|
| *Health-related medical scenario*<br>A medical doctor at one of the hospitals most affected by the Covid-19 health emergency finds himself having to choose who to prioritize for treatment between two elderly patients, both in their 80s. The first is a hypertensive patient, he is a widower but his daughter, suffering from a chronic illness, has always taken good care of him and now anxiously awaits his news; the second, on the other hand, is a diabetic. | *If you were the doctor, which of the two patients would you have given priority to?*<br>a) I would have given priority to the first patient since, in addition to being chronically ill, he has a daughter who is also ill and needs him (*emotional connotation*)<br>b) I would have given precedence to the first patient since hypertension is a more serious disease than diabetes: his situation is critical and he has already suffered greatly (*emotional connotation*)<br>c) I would have given precedence to the second patient because diabetes is at less risk of complications than severe hypertension, and therefore has a better chance of being saved (*cognitive connotation*)<br>d) I would have given priority to the second patient because if stabilized immediately, he could be discharged quickly, freeing up a place for more serious patients (*cognitive connotation*) |
