# Supplementary material for: Emotional and Cognitive “Route” in Decision-Making Process: The Relationship between Executive Functions, Psychophysiological Correlates, Decisional Styles, and Personality
Source: Brain Sci. 2024 Jul 22;14(7):734. doi: 10.3390/brainsci14070734 (PMC11274958; doi:10.3390/brainsci14070734)
Supplement: Supplementary file 1 [file brainsci-14-00734-s001.zip › SupplMatS2_BrainSciences.pdf]

Supplementary Material S2

Additional methodological notes on score calculations

ECP_i calculation:

In this first equation, $Resp_{Emo\text{-}Org}$ stands for decile response scores at emotion-connoted statements for organizational scenarios, $Resp_{Emo\text{-}Med}$ stands for decile response scores at emotion-connoted statements for medical scenarios, $RT_{Emo\text{-}Org}$ stands for mean RTs converted in a decile scale for emotion-connoted statements in organizational scenarios, and $RT_{Emo\text{-}Med}$ stands for mean RTs converted in a decile scale for emotion-connoted statements in medical scenarios.

CCP_i calculation:

In this second equation, $Resp_{Cog\text{-}Org}$ stands for decile response scores at cognition-connoted statements for organizational scenarios, $Resp_{Cog\text{-}Med}$ stands for decile response scores at cognition-connoted statements for medical scenarios, $RT_{Cog\text{-}Org}$ stands for mean RTs converted in a decile scale for cognition-connoted statements in organizational scenarios, and $RT_{Cog\text{-}Med}$ stands for mean RTs converted in a decile scale for cognition-connoted statements in medical scenarios.

TR_{PSD} calculation:

In this third equation, TR_{PSD} represents task-related variations in EEG power values for each frequency band and each electrode site, PSD_{task} represents EEG power values during the task for each frequency band and each electrode site, and $PSD_{open\text{-}bl}$ represents EEG power values in eye-open baseline for each frequency band and each electrode site.

TR_{AI} calculation:

In this fouth equation, TR_{AI} represents task-related changes in the autonomic measure (SCL, SCR, HR, or HRV), AI_{task} represents the autonomic activity during the task for each measure, and $AI_{open\text{-}bl}$ represents the autonomic activity in eye-open baseline for each measure.
